# Endoscopic Gallbladder Drainage: A Comprehensive Review on Indications, Techniques, and Future Perspectives

**DOI:** 10.3390/medicina60040633

**Published:** 2024-04-14

**Authors:** Edoardo Troncone, Rosa Amendola, Alessandro Moscardelli, Elena De Cristofaro, Pasquale De Vico, Omero Alessandro Paoluzi, Giovanni Monteleone, Manuel Perez-Miranda, Giovanna Del Vecchio Blanco

**Affiliations:** 1 Department of Systems Medicine, University of Rome “Tor Vergata”, 00133 Rome, Italy; 2 Department of Anaesthesia, University of Rome “Tor Vergata”, 00133 Rome, Italy; 3 Department of Gastroenterology and Hepatology, University Hospital Rio Hortega, 47012 Valladolid, Spain

**Keywords:** acute cholecystitis, EUS-guided drainage, interventional EUS, therapeutic EUS, ERCP, cholecystostomy, cholecystectomy, biliary obstruction

## Abstract

In recent years, therapeutic endoscopy has become a fundamental tool in the management of gallbladder diseases in light of its minimal invasiveness, high clinical efficacy, and good safety profile. Both endoscopic transpapillary gallbladder drainage (TGBD) and endoscopic ultrasound (EUS)-guided gallbladder drainage (EUS-GBD) provide effective internal drainage in patients with acute cholecystitis unfit for cholecystectomy, avoiding the drawbacks of external percutaneous gallbladder drainage (PGBD). The availability of dedicated lumen-apposing metal stents (LAMS) for EUS-guided transluminal interventions contributed to the expansion of endoscopic therapies for acute cholecystitis, making endoscopic gallbladder drainage easier, faster, and hence more widely available. Moreover, EUS-GBD with LAMS opened the possibility of several cholecystoscopy-guided interventions, such as gallstone lithotripsy and clearance. Finally, EUS-GBD has also been proposed as a rescue drainage modality in malignant biliary obstruction after failure of standard techniques, with encouraging results. In this review, we will describe the TBGD and EUS-GBD techniques, and we will discuss the available data on clinical efficacy in different settings in comparison with PGBD. Finally, we will comment on the future perspectives of EUS-GBD, discussing the areas of uncertainty in which new data are more strongly awaited.

## 1. Introduction: Rationale for Endoscopic Gallbladder Drainage

Gallbladder disorders are a heterogeneous group of diseases frequently encountered in the general population, with a significant impact on healthcare costs [1,2,3]. Acute cholecystitis and symptomatic gallstones are the most frequent clinical presentations. Surgical cholecystectomy has, for decades, been the mainstay of treatment of these conditions; currently, the minimally invasive laparoscopic approach still represents the reference standard treatment for acute cholecystitis [3,4,5,6]. However, surgery is not equally safe in very ill, frail, or complex patients with significant comorbidities, which are, accordingly, deemed high-surgical-risk candidates for cholecystectomy. Indeed, cholecystectomy in this group may lead to high morbidity and mortality rates [7,8,9]. Such patients benefit from minimally invasive drainage, which is aimed at controlling the local source of infection/inflammation. According to the 2018 Tokyo Guidelines, the treatment of acute cholecystitis should be planned based on the severity of the cholecystitis, the American Society of Anesthesiologist (ASA) status, and patient comorbidities, evaluated through the Charlson Comorbidity Index (CCI) [10]. Patients with grade 3 cholecystitis, who present signs of organ failure, and patients with grade 2 cholecystitis and high ASA and CCI scores are at high surgical risk; for these two patient subsets, surgery should best be avoided. Patients unfit for surgery are candidates for minimally invasive gallbladder drainage (GBD) whenever medical therapy fails to control local and systemic inflammation [7,10]. The placement of a percutaneous gallbladder drainage (PGBD) tube is a time-honored and effective strategy to control local and systemic infection, minimizing surgical-related morbidity [7,11,12]. Despite the wide availability and the excellent technical and clinical outcomes, PGBD is burdened by a high reintervention rate and a negative impact on patients’ quality of life; PGBD cannot be considered an effective permanent treatment for patients who will never undergo surgery [13,14,15]. Surprisingly, a randomized clinical trial (RCT) comparing laparoscopic cholecystectomy and PGBD (CHOCOLATE trial) reported a significantly higher rate of major complications, reinterventions, and recurrent biliary disease in the PGBD group compared to surgery, even in high-risk patients [16]. The limitations of PGBD encouraged the search for alternative drainage modalities, with endoscopic approaches to GBD emerging over time as widely used minimally invasive options.

GBD can be performed during endoscopic retrograde cholangiopancreatography (ERCP) by placing a transpapillary stent through the cystic duct (transpapillary gallbladder drainage, or TGBD). Such an approach is clinically effective but technically challenging as it needs selective cystic duct cannulation and stent placement, in addition to the complexity of a standard ERCP. In recent years, enormous progress in therapeutic endoscopic ultrasound (EUS) has boosted the possibility of performing endoscopic internal drainage and anastomoses, including endoscopic transmural drainage of the gallbladder. The availability of dedicated devices for EUS-guided drainage, namely, lumen-apposing metal stent (LAMS), has facilitated the endoscopic approach to several pancreatic-biliary diseases. Currently, EUS-GBD is increasingly being used in clinical practice in patients with acute cholecystitis deemed unfit for surgery. Moreover, EUS-GBD has also been proposed as an alternative method of biliary drainage in malignant biliary obstruction (MBO) when ERCP fails, further expanding the possible clinical applications of EUS-GBD.

In this review, we will summarize the evidence about endoscopic GBD, including EUS-guided and transpapillary drainage, focusing on technical considerations and clinical outcomes. Furthermore, we will discuss the place of these interventions in the therapeutic algorithm of gallbladder disease and biliary obstruction, highlighting unresolved issues in this evolving field.

## 2. Transpapillary Endoscopic Gallbladder Drainage: Technical Aspects

Endoscopically, the gallbladder is accessible either from the major papilla through the cystic duct during ERCP, or through transmural EUS-guided access across the gastric or duodenal walls. TGBD has the advantages of a “natural” route of drainage, without disrupting normal GI anatomy [17]. Additionally, the more widespread dissemination of ERCP compared to therapeutic EUS makes TGBD more widely available, since just ERCP expertise is required for TGBD.

**Figure 1:**
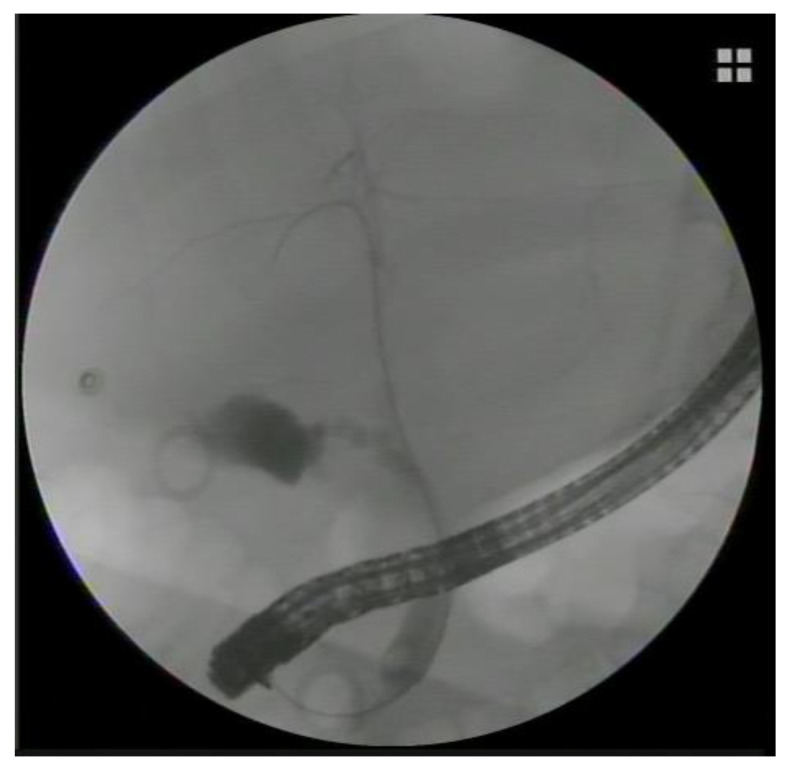
Fluoroscopic image of an endoscopic transpapillary gallbladder drainage. A 7 French, 12 cm plastic double pigtail stent was placed transpapillary during the ERCP in a patient with acute cholecystitis and common bile duct stones at high risk for surgery.

The first step of TGBD is selective cannulation of the cystic duct. This step is typically achieved with standard ERCP devices, such as a catheter or a sphincterotome with a 0.035-inch or a 0.025-inch guidewire. Occasionally, navigation through a tortuous or stenotic/angulated cystic duct may require a smaller caliber guidewire (i.e., 0.018 inch). In difficult cases, cannulation can be attempted with a second guidewire in parallel after occlusion of the hepatic duct proximal to the cystic duct take-off with a balloon [18]. Alternatively, cannulation can be achieved under direct view during cholangioscopy [19,20,21]. After selective cystic duct cannulation, usually, a small caliber biliary stent (5 to 7 French double pigtail stent) is advanced into the gallbladder (Figure 1). Alternatively, a nasogallbladder drainage tube can be placed instead of a plastic stent for temporary drainage [22,23]. In some cases, it may be necessary to dilate a stenosed cystic duct with mechanical dilators or a small caliber balloon to allow stent insertion, especially on rare occasions where more than one stent is planned to be placed [24]. However, placement of a second transcystic pigtail stent may be successful in less than half of attempted cases, according to a retrospective report on 51 TGBD patients [25]. Furthermore, the two-stent strategy did not confer any additional protection against acute cholecystitis recurrence compared to the placement of just one stent [25]. After stent placement, the follow-up strategy includes indefinite stenting in patients at high risk for reintervention, elective stent exchange, or stent removal before elective surgery, according to the patient’s general condition and surgical status (see below). Cannulation of the cystic duct may fail in the presence of obstructing stones or a neoplastic mass, anatomic factors that limit the success rate of TGBD. Even if successful gallstone dissolution via nasocystic catheters was historically reported, this approach was soon abandoned because of its unacceptable high rate of adverse events; no effective therapeutic interventions on gallstones through the cystic duct are currently available, which renders this approach less appealing for definitive treatment of gallstone-related diseases.

## 3. EUS-Guided Gallbladder Drainage: Technical Aspects

**Figure 2:**
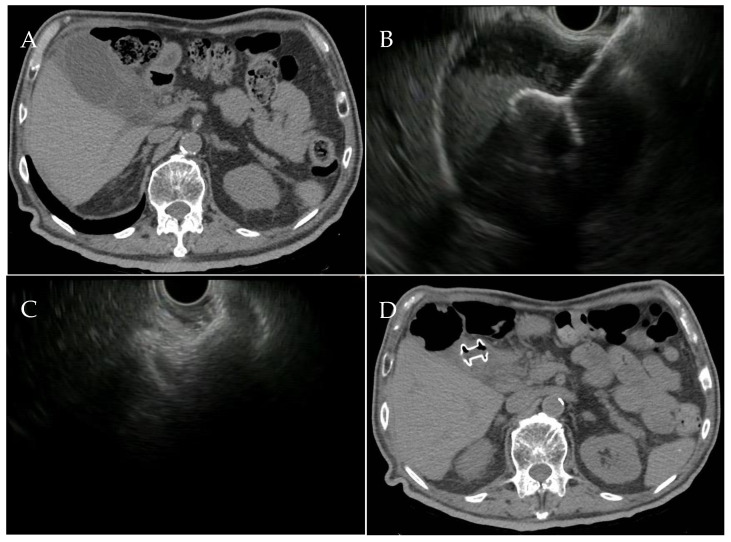
(**A**) CT scan image of an acute cholecystitis. (**B**) EUS images of the LAMS deployment and (**C**) the fully deployed LAMS into the gallbladder. (**D**) CT scan image showing the drained gallbladder with the LAMS in situ. CT: computed tomography; EUS: endoscopic ultrasound; LAMS: lumen-apposing metal stent.

The EUS-GBD technique is based on the creation of a transmural tract between the gastrointestinal tract and the gallbladder walls under EUS view. Traditionally, EUS-guided access to a target lumen was performed with a multi-step procedure, which involved puncture with a 19-gauge fine-needle aspiration (FNA) needle, insertion of a guidewire, dilation of the tract, and stent placement. In the beginning, luminal stents were adapted from ERCP to transmural drainages, such as plastic double pigtail stents (DPS) or self-expanding metal stents (SEMS). A systematic review with pooled analysis analyzed the technical and clinical outcomes of 166 EUS-GBD procedures performed with different types of stents (DPS, SEMS, and LAMS). The overall technical success, clinical success, and adverse events rates were 95.8, 93.4, and 12%, respectively, with good results also for non-dedicated devices [26]. However, the frequency of adverse events was 18.2% with DPS, 12.3% with SEMS, and 9.9% with LAMS. The use of plastic stents for transmural drainage may increase the risk of leakage due to the lack of sealing of the fistula. Conventional tubular SEMS may better seal the tract with their radial expansion force but might cause injury on contralateral walls and are prone to migration if not equipped with anti-migratory features [26]. A recent meta-analysis with meta-regression on 27 studies including 1004 EUS-GBD procedures reported the use of devices with an anti-migrating design as positively associated with clinical success and lower risk of adverse events [27]. LAMS for EUS-guided drainage represents a real game changer. With LAMS, which has a dedicated design for transmural drainage, EUS-guided drainage became a widely available procedure. These devices are self-expanding metal stents equipped with wide flanges that ensure a strong lumen-to-lumen apposition, anti-migratory properties, and sealing of the fresh transmural tract, minimizing the risk of extraluminal leakage. Initially used for EUS-guided drainage of peripancreatic fluid collections, indications have rapidly expanded to include EUS-guided biliary drainage, gallbladder drainage, and enteric anastomoses [28,29,30,31]. Currently, guidelines suggest the use of either LAMS or dedicated SEMS for EUS-GBD [32]. LAMS diameters range from 6 to 20 mm, with 10 mm and 15 mm being the most frequently used sizes. No comparative studies on different LAMS sizes are currently available; therefore, the choice of the size is usually dependent on the space within the gallbladder to accommodate the inner (or distal) flange of the LAMS, the size of gallstones, and the anticipated need of future re-interventions through peroral transluminal cholecystoscopy. Transluminal cholecystoscopy requires at least a 10 mm—and preferably a 15 mm—diameter LAMS. The addition of an electrocautery tip to the LAMS delivery system (Hot-LAMS) has enabled “free-hand” access to the target lumen, eliminating the drawbacks of multiple exchanges of the traditional multistep procedure. Therefore, EUS-GBD is most commonly performed with direct access of the Hot-LAMS delivery system into the gallbladder lumen, then followed by distal flange deployment under EUS, and eventually proximal flange deployment, either intra-channel or under endoscopic monitoring [33] (Figure 2). The traditional LAMS deployment technique over a previously placed guidewire may still be very helpful in difficult cases of sclerotic or poorly distended gallbladders, which carry an increased risk of stent misdeployment and may require salvage procedures. Currently, high-quality evidence comparing these two LAMS placement strategies is not available; the choice of one over another depends on the specific clinical situation and the operator’s personal preference. However, experience from EUS-guided enteric anastomoses highlights the risk of pushing away the target lumen when a guidewire has advanced into a mobile target, thus making subsequent LAMS placement more challenging. The endoscopist’s experience is crucial in choosing the best drainage technique, as well as in performing salvage maneuvers in case of unplanned procedural events. A further advantage of the direct LAMS placement technique is that the procedure can be performed without fluoroscopic guidance under complete EUS/endoscopic control. Nonetheless, rescue procedures in case of stent misdeployment or adverse events, as well as possible additional interventions such as coaxial DPS placement, may require fluoroscopic guidance. Hence, there is no uniform consensus about the optimal room set-up for EUS-GBD.

EUS-GBD can be performed through the gastric (cholecystogastrostomy) or duodenal (cholecystoduodenostomy) route. From a purely technical point of view, both sites are appropriate for stent placement as long as a safe EUS window, without intervening vessels, is found; the distance between the GI wall and the gallbladder should ideally be <10 mm, and not >15 mm in any case. However, the choice of the drainage site may impact clinical outcomes. Observational studies have suggested that the transgastric LAMS location is associated with an increased risk of stent obstruction due to food impaction and buried stent syndrome [32,34]. To improve long-term LAMS patency, the duodenal bulb should be preferred over the gastric antrum for EUS-GBD, whenever possible. However, no definitive evidence is yet available on transgastric vs. transduodenal LAMS patency rates, and preliminary data suggest that the outcomes across both subgroups are fairly comparable [35]. In this regard, it is unclear if the placement of coaxial DPS, which has been proposed in different clinical settings involving LAMS drainage, may reduce the risk of stent dysfunction [30,32,36,37,38]. On the other hand, transgastric EUS-GBD may be the preferred approach in patients with a possible future cholecystectomy, as the gastric fistula is perceived as less challenging to manage during laparoscopic cholecystectomy compared to the transduodenal fistula of a cholecystoduodenostomy (see below).

## 4. Gallbladder Drainage in Acute Cholecystitis: Percutaneous, Transpapillary, or EUS-Guided Approach?

**Figure 3:**
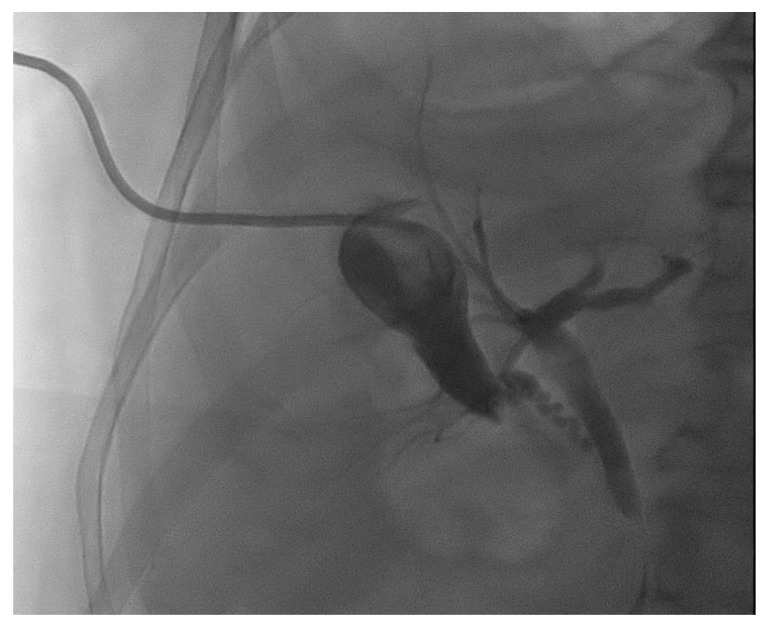
Fluoroscopic image of a percutaneous gallbladder drainage.

For decades, percutaneous gallbladder drainage (PGBD) has represented the most effective minimally invasive treatment for acute cholecystitis (Figure 3). As discussed above, PGBD is burdened by a high reintervention rate. Reintervention is often secondary to tube-related issues such as blockage requiring exchange or spontaneous tube dislodgement. On the other hand, PGBD patients who have their tubes removed and do not undergo cholecystectomy exhibit a high rate of acute cholecystitis recurrence [13,14,15]. In contrast, endoscopic GBD offers a minimally invasive yet definitive treatment option if the patient remains a non-surgical candidate, without the negative impact on the quality of life of percutaneous external tubes [15,16,39]. To compare technical and clinical outcomes of different drainage approaches in high-risk surgical patients with acute cholecystitis, Mohan and colleagues conducted a large meta-analysis including 82 studies and 1223 patients, 557 patients, and 13 351 patients treated by TGBD, EUS-GBD, and PGBD, respectively [40]. The authors reported a pooled technical and clinical successes for TGBD of 83% (95% confidence interval [CI]: 80.1–85.5) and 88.1% (95%CI: 83.6–91.4), respectively; for EUS-GBD, 95.3% (95%CI: 92.8–96.9) and 96.7% (95%CI: 94.0–98.2), respectively; and for PGBD, 98.7% (95%CI: 98.0–99.1) and 89.3% (95%CI: 86.6–91.5), respectively. These results underscore the significantly lower technical and clinical success rate of TGBD compared to the other two approaches, a fact explained by the difficulties involved in selective cannulation of the cystic duct discussed above. Moreover, EUS-GBD performed significantly better than PGBD, with a very high technical success rate. The optimal results of EUS-GBD can be attributed to ease of placement and enhanced performance afforded by the specific design of LAMS, which provides stable drainage through a wide lumen diameter. Interestingly, the same meta-analysis reported a significantly lower rate of acute cholecystitis recurrence for either endoscopic strategy compared to the percutaneous approach (4.6%; 95%CI: 2.8–7.4 with TGBD; 4.2%; 95%CI: 2.4–7.4 with EUS-GBD; 10.8%; 95%CI: 8.3–13.9 with PGBD), further confirming that PGBD is sub-optimal for the long-term management of acute cholecystitis. A systematic review with network meta-analysis from Podboy and colleagues showed similar results, with a higher likelihood of technical success (EUS-GBD vs. PGBD vs. TGBD: 2.00 vs. 1.02 vs. 2.98) and clinical success (EUS-GBD vs. PGBD vs. TGBD: 1.48 vs. 1.55 vs. 2.98) for EUS-GBD and PGBD compared to TGBD. Once again, PGBD was associated with a higher risk of re-interventions and unplanned re-admissions, while EUS-GBD had the lowest risk of cholecystitis recurrence (EUS-GBD vs. PGBD vs. TGBD: 1.089 vs. 2.02 vs. 2.891) [41]. Most of the studies included in the meta-analyses are limited by their retrospective design and thus characterized by heterogeneity in the population and the EUS-guided approaches included. Therefore, a recent randomized controlled trial (RCT) conducted by Teoh and colleagues [42] was welcomed. In this study, patients with acute cholecystitis at very high risk for cholecystectomy were randomized to EUS-GBD (n = 39) or PGBD (n = 40). The primary outcome was the 1-year adverse event rate, and secondary outcomes included technical and clinical success, 30-day adverse events, pain scores, unplanned readmissions, re-interventions, and mortality. The EUS-GBD was performed using the cautery-enhanced LAMS “Hot AXIOS” (Boston Scientific Medical Corporation, Marlborough, MA, USA), which is the most widely used Hot-LAMS for EUS-guided interventions so far, with either the “free-hand” puncture or over-the-wire techniques. As expected, technical and clinical success rates were similar between the two groups (97.4% vs. 100%, *p* = 0.494; 92.3% vs. 92.5%, *p* = 1, respectively). However, EUS-GBD outperformed PGBD in the primary outcome (1-year adverse event rate: 10 (25.6%) vs. 31 (77.5%), *p* < 0.001) and in most secondary outcomes, such as 30-day adverse events (5 (12.8%) vs. 19 (47.5%), *p* = 0.010), re-interventions after 30 days (1/39 (2.6%) vs. 12/40 (30%), *p* = 0.001), unplanned readmissions (6/39 (15.4%) vs. 20/40 (50%), *p* = 0.002), and recurrence of cholecystitis (1/39 (2.6%) vs. 8/40 (20%), *p* = 0.029). Notably, a relevant proportion of adverse events in the PGBD group was related to the drainage tube, such as tube dislodgement. Taken together, these data have convincingly placed EUS-GBD as the preferred drainage technique in patients with acute cholecystitis at high risk for cholecystectomy, when the expertise is available [32,43]. Recently, good technical and clinical outcomes were reported for EUS-GBD using a different LAMS with a lower lumen opposing force, the Spaxus stent, which is available with both standard and cautery-tipped delivery systems (Spaxus/Hot-Spaxus, Taewoong Medical Co., Gimpo, Republic of Korea) [44,45].

**Figure 4:**
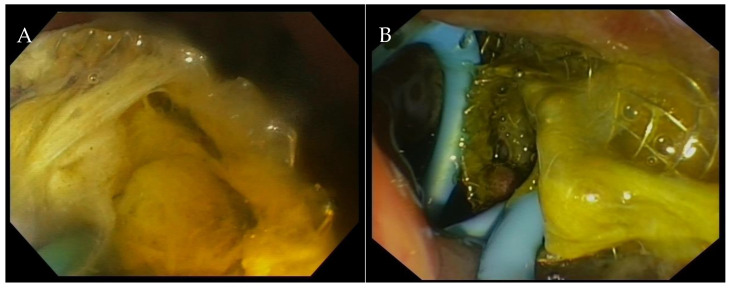
(**A**) Endoscopic image of an obstructed LAMS. (**B**) After stent cleansing, a coaxial double pigtail plastic stent was placed to avoid further obstruction. LAMS: lumen-apposing metal stent.

Both TGBD and EUS-GBD have proven to be safe procedures, with acceptable rates of adverse events. TGBD is burdened with the classical ERCP-related risks (i.e., bleeding, pancreatitis, perforation). The EUS-GBD adverse event rate is about 4–22%, including bleeding, stent misdeployment, perforation, pneumoperitoneum, bile leak, buried stent, and stent occlusion with recurrent cholecystitis [38,39,40] (Figure 4). Most such events can effectively be treated endoscopically. For instance, stent misdeployment with perforation limited to the gastrointestinal wall can be closed with clips. A meta-analysis including 546 EUS-GBD reported an overall risk of adverse events of 12.4%, which was not statistically different compared to TGBD (9.6%) and PGBD (15.1%) [40]. However, the rate of bleeding and perforation was significantly higher for EUS-GBD, compared to PGBD and TGBD (4.3% vs. 2% vs. 1.9%, *p* =0.02, for bleeding; 3.7% vs. 2% vs. 2%, *p* = 0.04, for perforation). EUS-GBD should be considered a high-risk procedure in terms of bleeding; therefore, anti-thrombotic drugs should be managed accordingly, even though successful and uneventful EUS-GBD has been anecdotally reported as a last resource in patients with coagulopathy or on anticoagulation therapy [46,47]. On the other hand, PGBD has an increased risk of stent migration/dislodgement and a higher overall adverse events risk according to another recent meta-analysis [40,48].

**Figure 5:**
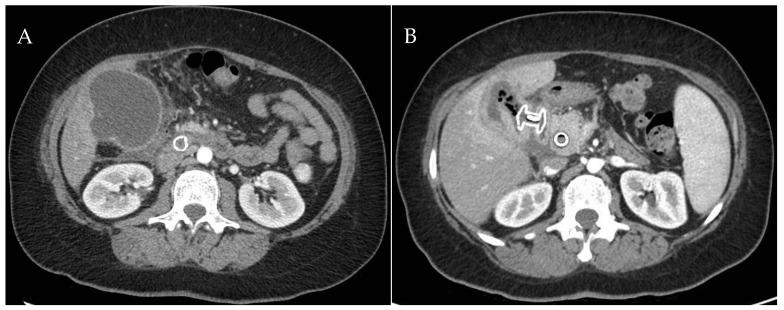
(**A**) CT scan image of acute cholecystitis after biliary SEMS placement in a jaundiced patient with pancreatic head cancer. (**B**) Drained gallbladder after LAMS placement. CT: computed tomography; SEMS: self-expanding metal stent; LAMS: lumen-apposing metal stent.

Do these data suggest that one drainage strategy is clearly superior to others and that it should always be preferred? Despite the excellent outcomes of EUS-GBD, which seems to be the most effective approach for minimally invasive management of acute cholecystitis, the answer is still no, as many variables have to be taken into account when treating such complex patients. First of all, endoscopic drainage, either TGBD or EUS-GBD, requires that the patient be fit enough for sedation. Therefore, PGBD is still the best and only feasible approach when the patient is too unstable to undergo sedation and therapeutic endoscopy given that PCBD can be performed under minimal or no sedation and local anesthesia. Moreover, PGBD is still the best choice in patients with gallbladder perforation [38]. Additionally, the role of EUS-GBD in operable patients is still debated (see below). When the possibility of a cholecystectomy after clinical stabilization and sepsis control has not been completely ruled out, drainage strategies that do not alter the anatomy, namely, PGBD or TGBD, have the potential to make surgery less challenging in the future, and that is why these strategies are currently favored over EUS-GBD, at least until more data confirm preliminary data suggesting that cholecystectomy can be safely performed after EUS-GBD with LAMS [49]. For instance, a patient with an undecided surgical plan after sepsis resolution and concomitant indication for ERCP is a good candidate for TGBD. Furthermore, TGBD could also be an effective and safe approach in patients with ascites, in whom both PGBD and EUS-GBD are more difficult and burdened with higher risks, or in patients with coagulopathy/anti-coagulant use, since a transpapillary stent does not require sphincterotomy for placement. On the other hand, EUS-GBD is the only possible endoscopic approach when the papilla is not reachable, such as in case of post-surgical anatomy or duodenal obstruction, or when the cystic duct is inaccessible (i.e., SEMS in place, neoplastic obstruction) [50,51] (Figure 5). Finally, EUS-GBD is the only approach that allows for subsequent safe and effective therapeutic interventions on the gallbladder, such as cholecystoscopy with stone extraction; therefore, EUS-GBD could be favored if such a need can be anticipated (e.g., patients with a large stone burden).

The picture that emerges is that we have various strategies with different strengths and weaknesses, which meet the needs of different patient subsets in different clinical scenarios. Therefore, there is not a single approach that fits every possible case well. While EUS-GBD is emerging as the best strategy for many cases due to its short- and long-term efficacy, it is often helpful to evaluate the patients in a multidisciplinary team, involving surgeons and anesthesiologists, to share decision-making and to propose the best management pathway to the patient.

## 5. Therapeutic Strategies after a Successful Endoscopic Gallbladder Drainage: What Is Next?

The possible strategies after a successful endoscopic GBD depend on several factors, which include the type of drainage performed; the general condition of the patient, which, in turn, may determine if additional endoscopic procedures and sedation can be tolerated; and possible reversal from the status “unfit for surgery” to the surgical candidate, and vice versa. In the case of very frail patients, additional endoscopic procedures may best be avoided. In these cases, the index GBD procedure may be intended as a definitive treatment. But what is the long-term outcome in these cases? A retrospective monocentric study reported the outcome of 234 patients who underwent TGBD with a single 7 French/15 cm DPS, without scheduled stent exchanges, with a median follow-up period of 564 days (range 200–3001 days). The authors reported a biliary event-free rate of 99% at 6 months, 92% at 1 year, and 76% at ≥2 years, supporting the use of TGBD as definitive treatment in high-risk patients due to a favorable biliary event-free rate at 2 years [52]. Another retrospective study including 49 TGBD reported a long-term clinical success (i.e., no acute cholecystitis recurrence; death or re-interventions at 6 months) of 95.9% (47/49) [25]. Mean follow-up was 453 days (range, 18–1879), and placement of a single stent was associated with a higher number of repeated procedures (*p* = 0.045) [25]. Inoue and colleagues performed a retrospective study with propensity score analysis to compare the long-term outcome of TGBD (n = 90) and EUS-GBD (n = 90) in high-risk patients with acute cholecystitis [53]. In this study, EUS-GBD was performed with plastic stents. Interestingly, the rate of acute cholecystitis recurrence was similar between the two groups (3.8% vs. 3.0%, *p* = 1.000). However, the overall late AE rate was significantly lower with EUS-GBD compared to TGBD (5.0% vs. 16.4%, *p* = 0.029), and this was secondary to the high rate of cholangitis in TGBD group. The authors hypothesized that the long-term presence of a stent in the bile duct may cause cholestasis and duodenal reflux, resulting in cholangitis and liver abscesses [53,54]. Therefore, these findings should be taken into account when planning a long-term GBD drainage without scheduled stent exchange. EUS-GBD, which is usually performed with a larger diameter stent (i.e., 10–15 mm), can theoretically provide more stable long-term drainage compared to the smaller caliber stents usually placed for TGBD. A retrospective analysis of EUS-GBD with LAMS and long stent dwell time (1 year) did not report significant stent-related adverse events and a cumulative 4.5% frequency of new hospital admissions for gallstone-related disease [55]. Similar results were reported in a large prospective Spanish study including 82 EUS-GBD, of which 45 completed a 1-year follow-up, that reported an overall 1-year cumulative risk of recurrent biliary events of 9.7% (4.1–21.8%) and a 1-year risk of adverse events and severe adverse events of 18.8% (11–31.2%) and 7.9% (3.3–18.2%), respectively [56]. A retrospective study including 51 patients who underwent EUS-GBD with LAMS with 3 years follow-up and no scheduled interventions reported adverse events occurring in 18%, 20%, and 26% of patients in the first, second, and third years, respectively [57]. The overall acute cholecystitis recurrence was 4%. Interestingly, symptomatic LAMS-related adverse events were associated with a gastric location of the stent compared with a duodenal location (66.7% vs. 12.5%; *p* = 0.03) [57]. Taken together, these data suggest that EUS-GBD with LAMS could serve as a long-term therapeutic strategy in patients unfit for further interventions, with an acceptable risk of adverse events and biliary event recurrence. However, in patients who can undergo additional endoscopic procedures, it is commonly recommended to complete stone clearance through peroral cholecystoscopy 2–4 weeks after the index procedure and to remove the LAMS, leaving in place a DPS across the fistulous track to avoid the risk of LAMS-related adverse events [32,42,43,58]. Despite guideline endorsement of this second-look approach, there is no concrete evidence of its superiority over the alternative strategy of LAMS as “destination therapy” for EUS-GBD.

## 6. EUS-Guided Gallbladder Drainage in Possible Surgical Candidates

Some patients originally deemed unfit for surgery may have their surgical risk reduced after treating the sepsis and stabilizing their systemic comorbidities. The role of EUS-GBD as a bridge-to-surgery is still undefined, due to the concerns about the surgical management of the choelcystogastric or, particularly, the cholecystoduodenal fistula, that may hinder a successful laparoscopic intervention. A multicenter retrospective analysis compared the outcome of cholecystectomy after EUS-BGD (n = 46) or PGBD (n = 93) [59]. In this study, all LAMS were removed before surgery, and the fistula was closed with an over-the-scope clip (OTSC). The rate of open cholecystectomy was not statistically significantly different between groups (EUS-GBD 11/46, 24%; PGBD 8/93, 9%; *p* = 0.068), as well as the rate of conversion from laparoscopic to open (EUS-GBD 5/46, 11%; PGBD 18/93, 19% (*p* = 0.2324). These findings pointed out the feasibility of laparoscopic cholecystectomy after EUS-GBD. However, it should be noted that most of EUS-GBD had been performed transgastrically (80.4%). Despite that, about 35% of cases in the EUS-GBD group were finally managed with open cholecystectomy [59]. Recently, Bang and colleagues reported 25 cases of cholecystectomy after EUS-GBD [60]. In three cases of transgastric EUS-GBD (12%), a minimally invasive approach (robotic or laparoscopic cholecystectomy) was unsuccessful due to the presence of pericholecystic adhesions and/or cholecystogastric fistula, which necessitated conversion to open or subtotal cholecystectomy. Based on this limited retrospective experience, the authors strongly felt that EUS-GBD should only be used in “never-surgery” patients, instead of high-risk patients. However, such a restrictive recommendation for the use of EUS-GBD contradicts data from larger prospective and retrospective studies and may lead to high-surgical risk patients with acute cholecystitis receiving suboptimal drainage treatment options. Even if the interpretation of the evidence accrued so far may be insufficient for definitive conclusions and lend itself to controversial interpretations, local expertise and treatment algorithms should be developed to define optimal treatment pathways for predefined clinical scenarios. Multidisciplinary protocols shared with surgeons, anesthesiologists, and radiologists are strongly suggested in centers that treat patients with acute cholecystitis. Finally, it is also possible that a patient originally deemed fit for surgery who initially underwent PGBD, is subsequently and definitely deemed unfit for surgery. In such cases, the conversion of the PGBD to internal drainage can represent a good long-term strategy to prevent recurrence and avoid the drawbacks of the percutaneous drainage tube. PGBD conversion to either TBGD or EUS-GBD has been described as feasible and effective [61,62,63,64]. The steps of PGBD-to-EUS-GBD conversion with LAMS are the same as primary drainage, with the key difference that the gallbladder more often presents with a collapsed lumen and thickened walls, which carry an increased technical difficulty for LAMS placement [61]. Conversely, a stable patient with a gallbladder containing clean saline solution and not pus decreases the risks of adverse events associated with these technical difficulties encountered during drainage. More data are awaited on this topic.

## 7. EUS–Gallbladder Drainage as a Rescue Strategy in Malignant Biliary Obstruction

In recent years, EUS-guided biliary drainage (EUS-BD) has been increasingly used as an alternative to percutaneous transhepatic biliary drainage (PTBD) in malignant biliary obstruction (MBO) after ERCP failure because of the high efficacy, safety, and lower rate of re-interventions [65,66,67]. The most-used EUS-BD modalities are EUS-guided choledochoduodenostomy (EUS-CDS) and hepaticogastrostomy (EUS-HGS), which are very effective but require significant expertise and are limited by the caliber of the target duct [68,69,70,71]. In this setting, EUS-GBD has been proposed as a rescue drainage technique after ERCP failure if alternative EUS-BD techniques are not feasible or if operator expertise is limited. The advantages rely on the larger size of the target lumen, as the gallbladder is usually very well distended in the setting of distal MBO and can be “easily” targeted under EUS. With this technique, the entire biliary tree is internally drained into the GI lumen through the cystic duct, the patency of which is a pre-requisite for EUS-GBD as a drainage option in MBO. Several retrospective studies have reported the feasibility and effectiveness of EUS-GBD in this setting [72,73,74,75,76] (Table 1). In 2021, a retrospective study by Issa and colleagues reported the outcome of 28 EUS-GBD in distal MBO after failed ERCP [77]. The most frequently used stent for transmural drainage was a LAMS (26/28, 93%), but in two cases (2/28, 7%) a SEMS was placed. Technical success was achieved in 100%, and clinical success was achieved in 26 patients (92.6%), which was defined as a significant decrease in serum bilirubin within two weeks of the procedure. The overall adverse event rate was 17.8% (5/28), including three stent obstructions successfully managed endoscopically. The stent patency rate at 30 days from the procedure was 82%. Recently, a large retrospective Italian study included 48 EUS-GBD with LAMS, confirming the good technical and clinical results previously reported, with a technical and clinical success of 100% and 81.3%, respectively, and a mean total bilirubin reduction after 2 weeks of 66.5% [78]. The overall clinical success and adverse event rates reported in the meta-analysis are 85% and 13%, respectively [79,80]. EUS-GBD has also been investigated as a primary drainage technique in distal MBO. A prospective study including 37 consecutive patients reported 100% technical and clinical success, with 10.8% adverse events, including three stent disfunctions secondary to food impaction (n = 1) and cystic duct obstruction due to neoplastic progression (n = 2) [81]. Beyond the understandable enthusiasm with such good results, it should be recognized that long-term clinical data are still scarce, and we still do not know how reliable this route of drainage will be over time, especially in patients with a cystic duct that takes off very close to the neoplastic mass [82,83]. This is extremely important for patients who need chemotherapy, an increasing subgroup of pancreatic cancer patients [84]. Surgical cholecystoenterostomies have been performed for decades for jaundice palliation. According to this surgical experience, patients with surgical gallbladder drainage are at increased risk for re-interventions compared to patients who underwent surgical bile duct drainage [85,86,87]. EUS-GBD seems to be an effective immediate biliary decompression strategy in selected patients, but larger studies with longer follow-up data are required before this approach can be recommended as a primary biliary drainage option.

## 8. Unanswered Questions and Future Perspectives of EUS-Guided Gallbladder Drainage

Progress in gallbladder drainage procedures so far begs the question: how far can EUS-GBD go? The excellent clinical data started challenging the role of laparoscopic cholecystectomy as a definitive treatment in technically operable patients. In a retrospective study with propensity score analysis, Teoh and colleagues compared the outcome of EUS-GBD in high-risk patients with acute cholecystitis (n = 30) and with laparoscopic cholecystectomy (n = 30), including analysis at 1 year of follow-up [88]. Technical and clinical success was similar between the two groups. Strikingly, long-term outcomes were also comparable, including 30-day adverse events (4 [13.3%] vs. 4 [13.3%], *p* = 1), recurrent biliary events rates (3 [10%] vs. 3 [10%], *p* = 0.784), reinterventions (4 [13.3%] vs. 3 [10%], *p* = 1), and unplanned readmissions (3 [10%] vs. 3 [10%], *p* = 0.784). Notably, 1 year may be a too short a follow-up time to evaluate accurately the long-term effect of leaving a diseased gallbladder in situ, but these results are undoubtedly interesting [89].

New possibilities open up if we would consider eligible for EUS-GBD not only patients with acute cholecystitis but, in general, patients with the gallstone-related diseases (e.g., biliary pancreatitis, cholangitis, symptomatic cholelithiasis) who would benefit from a cholecystectomy to prevent recurrent biliary events. We already know that the population with gallstones-related diseases is becoming older, with complex comorbidities, and a significant proportion do not undergo elective cholecystectomy in the real world after a biliary event (e.g., symptomatic bile duct stones treated with an ERCP); this common real-life clinical scenario leaves patients at risk for recurrent events and further morbidity [90]. EUS-GBD could theoretically prevent such events, being also practicable during the same session of ERCP, but this is a topic for future investigations as the current evidence is limited; yet, as in other combined EUS and ERCP procedures, performing EUS-GBD in tandem with ERCP does not appear to increase procedural risks [91,92]. Interestingly, EUS-GBD, though only for prophylactic purposes, has already been investigated to prevent SEMS-related cholecystitis in distal MBO and cystic duct blockage by a tumor, with interesting results [51]. Another field of investigation would be the possible role of EUS-GBD in patients with cholecystitis or gallstones who are fit for surgery but at higher risk of conversion from a laparoscopic to laparotomic approach, with the attendant increased risk of morbidity [93,94,95].

The training in EUS-guided drainage procedures also requires further scrutiny. It is still unclear how many procedures are needed to achieve competency, and through which training path. An international multicenter retrospective study identified the endoscopist experience of fewer than 25 EUS-GBD as a predictor of unplanned procedural events and adverse events at 30 days [96]. However, the study also included multi-step devices for gallbladder drainage, for which the learning curve is expected to be longer compared to single-step Hot-LAMS. Additional studies are needed to establish the best training pathways and the optimal way to assess competency. In the meantime, training is mainly based on non-structured experiences with simulators, animal models, and mentorship at specialized high-volume centers.

## 9. Conclusions

The data discussed in this review underline that the endoscopic management of acute cholecystitis is feasible and effective, and it occupies a central position in a multidisciplinary group that takes care of patients with acute cholecystitis. In particular, high-quality evidence indicates that EUS-GBD is effective and safe as a drainage strategy in the acute setting, as well as long-term therapy in such patients, thanks to the high clinical success rate and low rate of re-interventions and cholecystitis recurrence. For these reasons, EUS-GBD deserves to be considered the first-line approach in most cases. However, a broad perspective is mandatory when managing patients with acute cholecystitis, as the drainage approach should also be chosen according to the mid-term and long-term clinical/surgical plans. In this regard, TGBD or PGBD may represent a better strategy in specific patient subgroups. These complementary approaches to gallbladder drainage should remain integrated in a complete therapeutic algorithm. EUS-GBD is also an effective rescue strategy to achieve biliary drainage in distal MBO in patients with patent cystic ducts after ERCP failure, but robust long-term data are still awaited. New indications for EUS-GBD still need validation through well-conducted studies and will be the object of future investigations.

## Figures and Tables

**Table 1:** Summary of the studies on EUS-guided gallbladder drainage for malignant biliary obstruction. SEMS: self-expanding metal stent; LAMS: lumen-apposing metal stent; BFMS: bi-flanged metal stent.

Author, Year [Reference]	Study Design	Indication	Number of Patients	Type of Stent	Technical Success	Clinical Success	Adverse Events	Stent Disfunction/Recurrent Jaundice	Follow-Up
Imai, 2016 [73]	Retrospective, single center	Rescue drainage	12	SEMS	12/12 (100%)	11/12 (91.7%)	2/12 (16.7%)	1 recurrent jaundice (8.3%)	105 days (median; 15–236 range)
Chang, 2019 [75]	Retrospective, single center	Rescue drainage	9	LAMS	9/9 (100%)	7/9 (77.8%)	0/9 (0%)	1 recurrent jaundice (11.1%)	130.7 days (mean)
Issa, 2020 [77]	Retrospective, multicenter	Rescue drainage	28	26 LAMS 2 SEMS	28/28 (100%)	26/28 (92.6%	5/28 (17.8%)	3 stent disfunction (10.7%)	33 months (median; 3–64 range)
Mangiavillano, 2023 [81]	Prospective, multicenter	Primary drainage	37	LAMS	37/37 (100%)	37/37 (100%)	4/37 (10.8%)	2 recurrent jaundice (5.4%) 1 stent difsunction (2.7%)	4 months (median; 1–8 range)
Binda, 2023 [78]	Retrospective, multicenter	Rescue drainage	48	45 LAMS 2 BFMS 1 other	48/48 (100%)	39/48 (81.3%)	5/48 (10.4%)	2 stent difsunction (4.2%)	122 ± 161 days (mean)
Debourdeau, 2024 [76]	Retrospective, multicenter	Rescue drainage	41	LAMS	41/41 (100%)	36/41 (87.8%)	4/41 (9.8%)	5 stent difsunction (12.2%)	5.2 months (1.21; 48.09) median

## Data Availability

Not applicable.
